# Spatio-temporal co-ordination of RhoA, Rac1 and Cdc42 activation during prototypical edge protrusion and retraction dynamics

**DOI:** 10.1038/srep21901

**Published:** 2016-02-25

**Authors:** Katrin Martin, Andreas Reimann, Rafael D. Fritz, Hyunryul Ryu, Noo Li Jeon, Olivier Pertz

**Affiliations:** 1 Dept. of Biomedicine, University of Basel, Mattenstrasse 28, 4058 Basel, Switzerland; 2 School of Mechanical and Aerospace Engineering, Seoul National University, Seoul, 151-742, Republic of Korea; 3 Institute of Advanced Machinery and Design, Seoul National University, Seoul, 151-742, Republic of Korea

## Abstract

The three canonical Rho GTPases RhoA, Rac1 and Cdc42 co-ordinate cytoskeletal dynamics. Recent studies indicate that all three Rho GTPases are activated at the leading edge of motile fibroblasts, where their activity fluctuates at subminute time and micrometer length scales. Here, we use a microfluidic chip to acutely manipulate fibroblast edge dynamics by applying pulses of platelet-derived growth factor (PDGF) or the Rho kinase inhibitor Y-27632 (which lowers contractility). This induces acute and robust membrane protrusion and retraction events, that exhibit stereotyped cytoskeletal dynamics, allowing us to fairly compare specific morphodynamic states across experiments. Using a novel Cdc42, as well as previously described, second generation RhoA and Rac1 biosensors, we observe distinct spatio-temporal signaling programs that involve all three Rho GTPases, during protrusion/retraction edge dynamics. Our results suggest that Rac1, Cdc42 and RhoA regulate different cytoskeletal and adhesion processes to fine tune the highly plastic edge protrusion/retraction dynamics that power cell motility.

Rho GTPases regulate the actin and adhesion dynamics that power cell motility. Initial models have proposed that Rac1 controls membrane protrusion, Cdc42 regulates filopodia and polarity, and RhoA promotes myosin contractility during tail retraction^1^. Measurements of spatio-temporal Rho GTPase activation dynamics using fluorescence resonance energy transfer (FRET)-based biosensors have challenged this classic model. All three Rho GTPases have been observed to be active at the leading edge of motile fibroblasts^2^,^3^,^4^,^5^,^6^. Furthermore, distinct Rho GTPase activity pools can be simultaneously activated at different subcellular locations - RhoA activity is associated with protrusion, tail retraction, and ruffling^5^; Cdc42 is activated in protrusions, filopodia, and at the Golgi^4^; Rac1 activity occurs during protrusion and ruffling^3^, but also controls invadopodia disassembly^7^. At the leading edge, the RhoA GTPase activation pattern can also depend on a specific stimulus – while fibronectin induces protrusions with high RhoA activity, platelet-derived growth factor (PDGF) leads to protrusions with reduced RhoA activity^5^. A correct understanding of spatio-temporal Rho GTPase activation dynamics therefore requires us to focus on context-dependent, specific subcellular morphodynamic processes, rather than on the entire cell^8^,^9^. Consistently, computational multiplexing of Rho GTPase activation dynamics has revealed precise, subminute time and micrometer length scale, co-ordination of RhoA, Rac1 and Cdc42 activities during edge protrusion/retraction cycles^10^. Specific patterns of RhoA, Rac1 and Cdc42 activation can also be observed at the leading edge of HT1080 fibrosarcoma cells^11^. Further, spatio-temporal signaling programs involving more than one Rho GTPase have been documented during Xenopus Laevis oocyte wounding^8^, or macropinocytosis^12^. While we start to unravel the diversity of spatio-temporal Rho GTPase signaling programs in different morphodynamic processes, their inherent complexity impedes a clear understanding of how they regulate cytoskeletal dynamics. Here, we take a reductionist approach, in which we study RhoA, Rac1 and Cdc42 GTPase activation dynamics during robust, prototypical edge protrusion/retraction events. This provides novel insight into how the three Rho GTPases co-operate to fine tune the cytoskeletal dynamics that power edge motility.

## Results

### Pulsed PDGF/Y-27632 application induces robust protrusion and retraction states

We used a flow-based, programmable microfluidic device (Fig. S1A) to manipulate edge dynamics through a 30 minutes pulse of growth factors/drugs that elicit cytoskeletal responses. REF52 rat embryonic fibroblasts were seeded in fibronectin-coated microfluidic devices for one hour, leading to an isotropic spreading state. Throughout all experiments, we co-imaged an Alexa 647-labeled dextran as a quality control for compound application/removal (Fig. S1B). The low level of flow in our microfluidic platform did not affect edge morphodynamics (Fig. S1C,D). In contrast, application of PDGF induced robust edge protrusion, while its removal led to immediate edge retraction (Fig. S1C,D, Movie S1). We focused on two distinct stimuli to manipulate cell edge dynamics. First, we studied PDGF because it induces robust protrusion with low RhoA activity^5^,^13^, raising the question which Rac1/Cdc42 activation dynamics could be associated with this morphodynamic state. Second, we used the Rho kinase-specific drug Y-27632^14^ that leads to protrusion by reducing intracellular contractility, and thus bypasses the need for an extracellular signal. This might provide insight into how an internal cytoskeletal state is interpreted to activate signaling responses.

**Figure 1:**
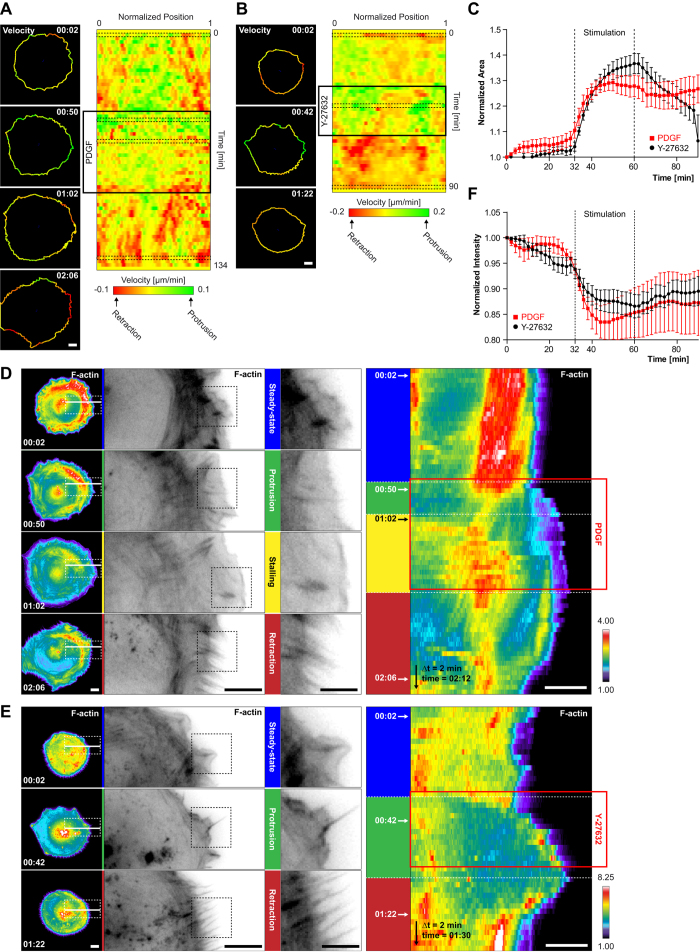
PDGF/Y-27632 pulse-induced edge and F-actin dynamics. (**A,B**) Quantification of edge dynamics upon PDGF (**A**) or Y-27632 (**B**) stimulation. The ADAPT Image J plugin^30^ was used to extract edge dynamics and velocities using the Lifeact-mCherry signal. Left panel: cell outlines display color-coded protrusion/retraction velocities (Time scale: hours:minutes). Right panel: velocity maps along the entire cell edge at normalized positions. Black dashed contours indicate specific cell outlines shown in the left panel. Color-code according to the scale bar. (**C**) Cell area dynamics in response to PDGF or Y-27632. Cell area was measured using ADAPT, and normalized to t = 0′. Average area ± s.e.m (PDGF: n = 12 cells, Y-27632: n = 13 cells). (**D,E**) F-actin dynamics during protrusion/retraction in response to PDGF (**D**) or Y-27632 (**E**). F-actin signals are color-coded for signal intensity or shown in inverted black and white (ibw) contrast. Color-code according to the scale bar. Left panel: F-actin signal (color-coded) of representative whole cells (left), as well as magnified insets (successively corresponding to the white and black dashed boxes). White solid lines represent ROIs used for kymograph analysis. Right panel: Kymograph analysis of F-actin dynamics during the PDGF/Y-27632 pulse. (**F**) Quantification of F-actin fluctuations. Whole cell lifeact-mCherry fluorescence intensity was averaged in response to a PDGF/Y-27632 pulse. Intensities were normalized to t = 0′. Average intensity ± s.e.m (PDGF: n = 5 cells; Y-27632: n = 5 cells) Scale bars: (**A,B,D,E**) 10 μm.

To get insight in the edge morphodynamics evoked by a PDGF or Y-27632 pulse, we tracked edge velocities during three distinct motility states: 1. 30 minutes before pulse application, 30 minutes of PDGF + Y-27632 pulse, 30 minutes immediately after PDGF/Y-27632/PDGF + Y-27632 washout. These three different edge motility states were timelapsed with a two minute time resolution. Before pulse application, a steady-state consisting of poorly protruding/retracting edges was observed (Fig. 1A,B). Previous analysis of steady-state edge dynamics had revealed cycles of protrusions and retractions, both with a persistence time of approximately 80 seconds^15^. Upon PDGF application, an almost isotropic burst of membrane protrusion persisted for a couple of minutes, until the edge stalled (Fig. 1A, Movie S1). PDGF withdrawal then led to edge retraction, again on a time scale of multiple minutes. In contrast, Y-27632 induced protrusions without a characteristic stalling phase (Fig. 1B, Movie S2). Edge retraction then immediately occurred after Y-27632 removal. These two distinct edge dynamics were also obvious when cell area was evaluated over time (Fig. 1C). Stimulation with both PDGF and Y-27632 recapitulated the Y-27632-evoked morphodynamics (Fig. S2A,B). These results indicate that our microfluidic platform induces robust protrusion and retraction events that occur on a different timescale than the edge dynamics observed at steady-state (e.g. multiple minutes rather than tens of seconds). Furthermore, distinct edge dynamics are evoked by the two stimuli. Upon combined PDGF + Y-27632 stimulation, identical edge motility behaviors as in response to Y-27632 alone are observed.

### Specific F-actin and adhesion dynamics correlate with prototypical edge protrusion/retraction events

We then evaluated cytoskeletal dynamics associated with steady, protrusion, stalling or retraction phases. Importantly, these dynamic states were not homogeneous across the cell edge. We therefore systematically gated our analysis on edge positions at which robust dynamic states occurred. In subsequent experiments, in which additional markers and FRET biosensors were imaged, we systematically monitored F-actin dynamics using Lifeact-mCherry, allowing for correlation across experiments. Different markers and biosensors were delivered using lentiviral and/or adenoviral vectors, enabling homogeneous and finely-tuned expression levels, not detrimental to cell function.

Analysis of F-actin dynamics during the steady-state phase revealed a prominent array of radial stress fibers^16^,^17^, indicating the presence of robust, actin retrograde flow in the lamella (Fig. 1D, Movie S1). Upon PDGF-induced membrane protrusion, this lamellar radial stress fiber network decompacted, and diminished in intensity. This was accompanied by formation of a robust lamellipodial network throughout, and directly at the cell edge (Fig. 1D). During the stalling phase, a radial stress fiber array then re-enforced about ten micrometers from the edge, while the edge lamellipodial network subsisted (Fig. 1D). PDGF withdrawal-induced retraction was characterized by lamellipodium disappearance, formation of edge retraction fibers, and condensation of the radial stress fiber lamellar network (Fig. 1D).

Y-27632 application led to immediate loss of the lamellar radial stress fibers, associated with strong protrusion propelled by lamellipodial networks (Fig. 1E, Movie S2). Edge retraction was again characterized by loss of the lamellipodial network, appearance of retraction fibers, as well as reformation of a lamellar radial stress fiber network. Both in response to PDGF or Y-27632 washout-triggered edge retraction, the intensity of the lamellar radial stress fibers network did however not recover to pre-pulse levels (Fig. 1F).

**Figure 2:**
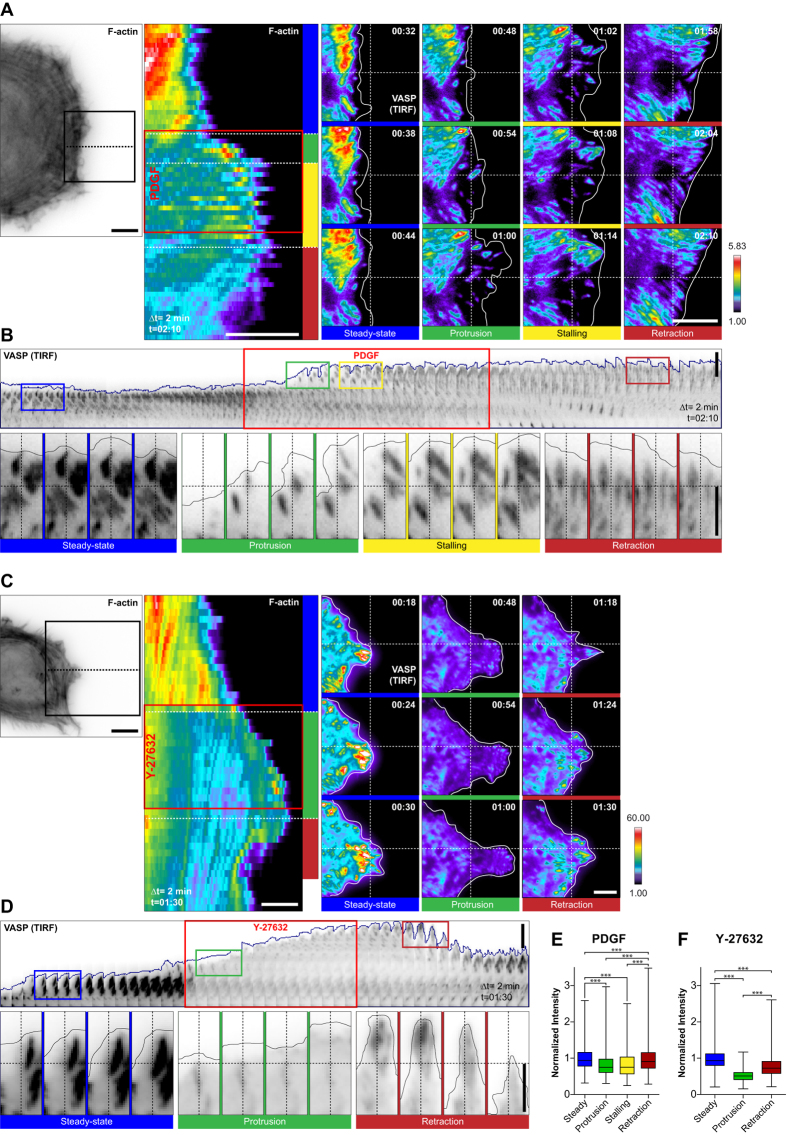
PDGF/Y-27632 pulse-induced adhesion dynamics. (**A–D**) Representative adhesion dynamics in response to a PDGF (**A,B**) or a Y-27632 (**C,D**) pulse. Cells expressing Lifeact-mCherry/VASP-GFP were imaged using epifluorescence (Lifeact) and TIRF (VASP) microscopy. In the insets, a dashed crosshair provides a virtual reference for visual inspection of the motile behavior of the adhesions. (**A,C**) Left panels depicts a micrograph (ibw contrast) and a Kymograph (color coded for fluorescence intensity) analysis of the F-actin signal along the dotted line. Right panels depict magnified insets of VASP-GFP signals in specific morphodynamic phases (from the solid line box in the left panel). VASP-GFP signal is color-coded for fluorescence intensity. Color-code according to the scale bar. Time scale: hours:minutes. (**B,D**) Kymograph analysis of adhesion dynamics in ibw contrast. Magnifications of selected insets from the kymograph (depicted by color-coded boxes corresponding to prototypical morphodynamic states) are also shown. Note that all the images have been scaled identically for a fair comparison of fluorescence intensities. (**E,F**) Quantification of adhesion fluorescence intensity in response to a PDGF (**E**) or a Y-27632 (**F**) pulse. Single adhesions were segmented using the Focal Adhesion Analysis Server (FAAS)^31^. Average fluorescence intensities were computed, and normalized to the pre-pulse, steady-state. Boxplots with median, interquartile (box) and 1.5 IQR (whiskers) range with of the fluorescence intensity distributions are shown (PDGF: n = 6 cells, 1300–1600 adhesions per condition; Y-27632: n = 7 cells, 650–1850 adhesions per condition; Non-gaussian distribution, Kruskal-Wallis test followed by Dunn’s multiple comparison post-test.; α = 0.05; ***p < 0.0001). Scale bars: (**A,C**) 10 μm. (**B,D**) 5 μm.

We then co-imaged F-actin and adhesion dynamics (VASP-GFP) in response to PDGF or Y-27632 pulses (Fig. 2A,C, left panels). At steady edges, elongated focal adhesions with characteristic centripetal sliding were observed. This is consistent with the robust actin retrograde flow that induces efficient conversion of focal complexes to focal adhesions (Fig. 2A,B, Movie S3). PDGF-evoked membrane protrusion led to formation of focal complexes that remained immobile with respect to the substrate, but then matured into focal adhesions during stalling. During edge retraction, these focal adhesions then increasingly elongated, and exhibited robust centripetal sliding.

Y-27632 led to immediate focal adhesions dissolution, leaving only small remnant adhesions (Fig. 2C,D, Movie S4). During retraction, focal adhesions were again formed from the remnant adhesion templates. Evaluation of VASP-GFP fluorescence intensities revealed a potent decrease in adhesion density in response to both PDGF and Y-27632, that however recovered to some extent during retraction, but never to pre-pulse levels (Fig. 2E,F).

Consistently with the finding of similar edge dynamics for both Y-27632, and PDGF + Y-27632 stimulation, identical F-actin (Fig. S2C,D) and adhesion dynamics (Fig. S2E,F) were again observed for both edge motility states. Together, these results indicate that specific F-actin and adhesion dynamics correlate with different edge motility states that are induced by PDGF, Y-27632, or combined application of PDGF + Y-27632.

### Construction of a FRET-based, Cdc42 activity biosensor

**Figure 3:**
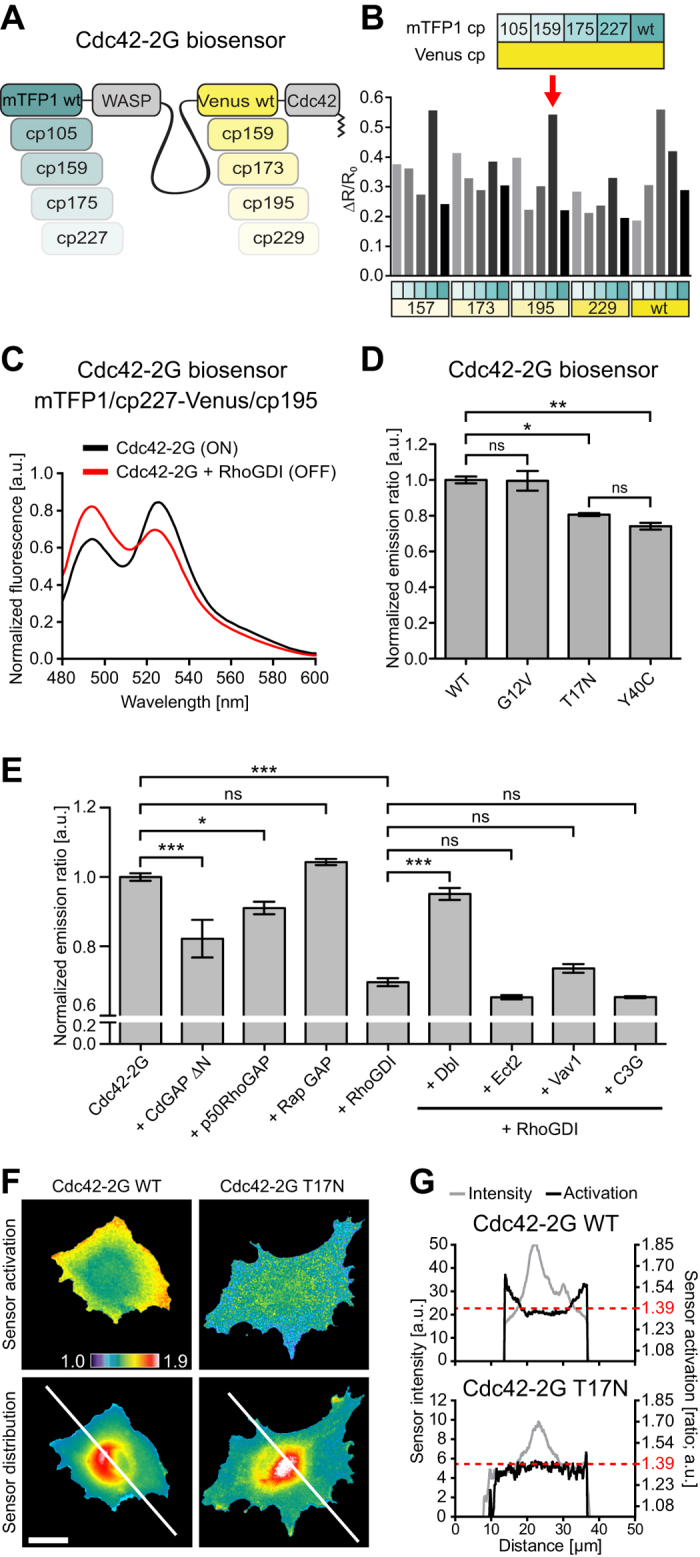
Construction and characterization of a genetically-encoded Cdc42 FRET biosensor. (**A**) Schematics of the 25 constructs of the Cdc42-2G biosensor library. (**B,C**) Fluorometry-based screening of the Cdc42 biosensor library. (**B**) A suspension of HEK293FT cells expressing Cdc42-2G alone (ON state) was measured. ΔR/R_0_ values represent changes in FRET efficiency between the ON or OFF states. Key indicates the specific mTFP1 and Venus mutants screened. Arrow indicates the selected biosensor. (**C**) Fluorescence spectrum of the selected Cdc42-2G biosensor. Spectrum is normalized by the area under the curve. (**D**) Evaluation of Cdc42-2G mutants. Indicated mutants were transfected and analyzed by fluorometry. n = 2 experiments. Gaussian distribution; Bonferroni’s multiple comparison test; α = 0.05; *P < 0.05; **P < 0.001. (**E**) Evaluation of Cdc42-2G response to upstream regulators. Transfected HEK293FT cells were analyzed in 96-well plates by high content microscopy. Average ER of multiple cells was calculated on a per-field of view basis. Bars represent average ± s.e.m. n ≥16 fields of view containing ≥100 cells each. Gaussian distribution; Bonferroni’s multiple comparison test; α = 0.05; *P < 0.05; ***P < 0.0001. (**F**) Evaluation of wild-type and T17N mutant Cdc42-2G biosensors in REF52 fibroblasts. ER (top) and biosensor distribution (donor channel; bottom) are shown. (**G**) Fluorescence intensity and ER profiles across lines shown in (**F**). ERs are scaled identically in both cells. Scale bar: (**F**) 10 μm.

To visualize Rac1, Cdc42, and RhoA activities, we used FRET-based biosensors. Multiple existing FRET-based biosensors report on RhoA, Rac1 and Cdc42 activation dynamics^2^,^4^,^5^,^6^,^7^,^13^,^18^ with different levels of sensitivity. However, some of these probes are constitutively tethered to the plasma-membrane^2^,^6^, and therefore cannot integrate any level of regulation exerted by Rho guanine nucleotide dissociation inhibitor (RhoGDI). At least for RhoA, this has been shown to be crucial to faithfully capture Rho GTPase activity patterns in time and space^13^. Using our cpFRET toolkit, which is based on a biosensor library with circularly permutated (cp) donor and acceptor fluorophores, we have previously constructed more sensitive, second generation, RhoA^13^, and Rac1^19^ biosensors (called RhoA-2G and Rac1-2G). We now report on the construction of a second generation, Cdc42-2G biosensor. For that purpose, we used a biosensor design that was successful in producing improved RhoA and Rac1 biosensors. This design includes wild type and four cps of the mTFP1 donor and Venus acceptor fluorophores, the WASP CRIB domain (amino-acids 201-314) to detect Cdc42 GTP-loading^4^, and Cdc42 at the C-terminus (Fig. 3A).

Screening of the library yielded three sensor constructs (mTFP1/cp227-Venus/cp157, mTFP1/cp227-Venus/cp195, mTFP1/cp175-Venus/wt) that displayed a robust change in emission ratio (ER) between ON and OFF states (Fig. 3B and S3A). We discarded the mTFP1/cp175-Venus/wt biosensor because mTFP1/cp175 displays a lower brightness than mTFP1/cp227^13^. Because the two remaining biosensors both contained mTFP1/cp227, we then arbitrarily chose the mTFP1/cp227-Venus/cp195 sensor variant for further investigation (Fig. S3B). Cdc42-2G showed a pronounced inversion of the donor and FRET emissions in the ON and OFF states (Fig. 3C), and appropriately responded to mutations that affect nucleotide loading (G12V and T17N) or effector interaction (Y40C)^20^ (Fig. 3D, S3C). Cdc42-2G responded to co-expression of Cdc42-competent Rho GTPase activating proteins (GAPs) such as a truncated form of CdGAP (CdGAP ∆N) or p50RhoGAP, but not the Rap1-specific RapGAP (Fig. 3E). Due to its specific design, Cdc42-2G also responded to RhoGDI. This drop of activity could then be rescued by co-expression of the Cdc42-competent guanine nucleotide exchange factor (GEF) Dbl, but not by non Cdc42-competent GEFs like Etc2 (RhoA-specific), Vav1 (Rac1-specific) or C3G (Rap1-specific). Finally, we analyzed the spatial Cdc42 activation patterns in living cells. As observed earlier^4^,^18^, Cdc42-2G detected broad Cdc42 activation at the cell edge (Fig. 3F). The Cdc42-2G T17N mutant, which retained a similar subcellular localization as Cdc42-2G, however yielded a flat emission ratio profile (Fig. 3F,G). This indicates that the Cdc42-2G sensor faithfully reflects Cdc42 activation pattern independently of cell geometry. A sequence map of Cdc42-2G is provided in Fig. S3D.

### Spatio-temporal Rho GTPase activation dynamics associated with prototypical protrusion/retraction events

**Figure 4:**
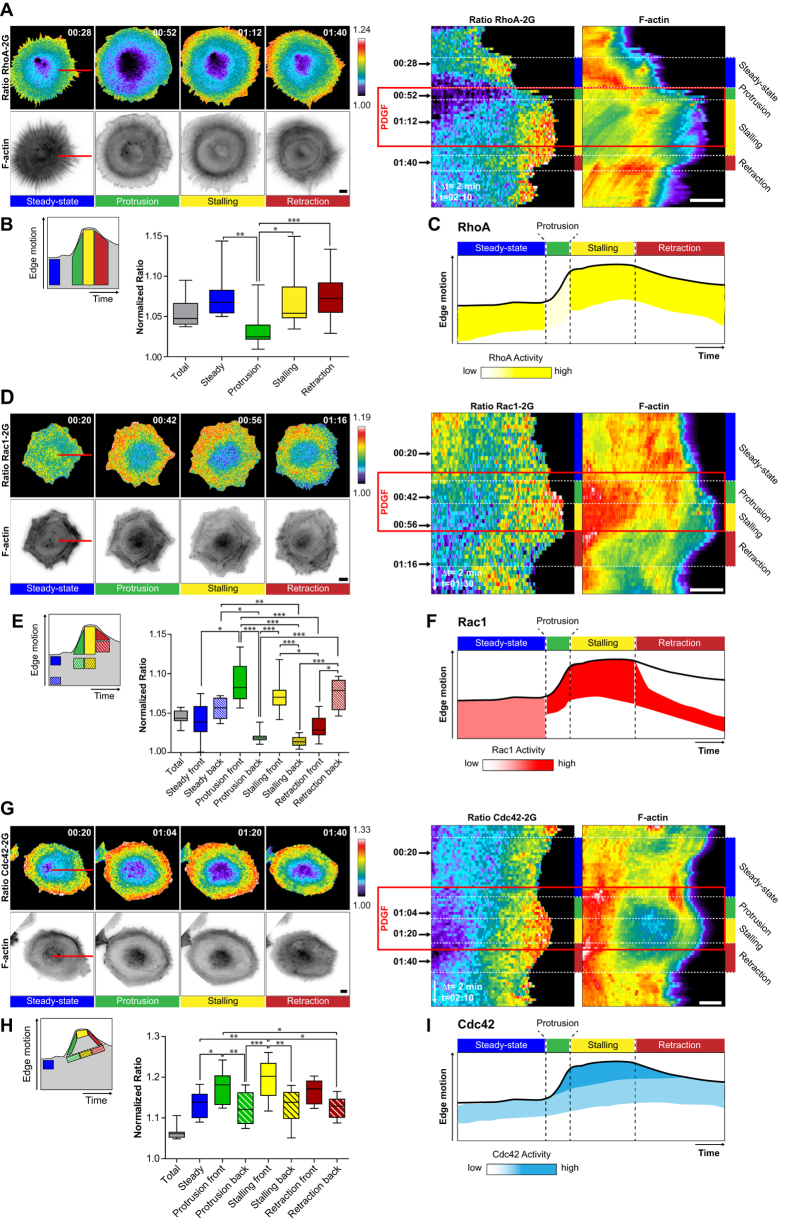
PDGF pulse-induced spatio-temporal Rho GTPase activation dynamics. (**A,D,G**) Spatio-temporal RhoA (**A**), Rac1 (**D**) and Cdc42 (**G**) activation simultaneously measured with F-actin dynamics. Left panel: Rho GTPase activation and F-actin signals representative of prototypical morphodynamic states. ERs are color-coded (top), and F-actin signals are shown in ibw contrast (bottom). Right panel: Kymograph analysis of ERs, and F-actin (color-coded for fluorescence intensity) along the red line shown in the micrographs. Time scale: hours:minutes. (**B,E,H**) Quantification of Rho GTPase signaling states. Left panel: schematic representation of kymograph ROIs that were used to quantify Rho GTPase activity. Right panel: Boxplots of ROI-averaged ERs, with median, interquartile (box) and 1.5 IQR (whiskers). ERs were normalized by the average of the 10% pixels with lowest ERs, representing basal Rho GTPase activity within the cell, enabling to compare ERs across different biosensors. Total averaged ERs (grey box) were not included in statistical analysis; RhoA: n = 5 cells, 8 to 21 measurements per dynamic event, Rac1: n = 6 cells, 10 to 11 measurements per dynamic event. Non-gaussian distribution; Kruskal-Wallis test followed by Dunn’s multiple comparison post-test; α = 0.05; *P < 0.05, **P < 0.001, ***P < 0.0001, non-indicated pairs show no significance; Cdc42: n = 5 cells 8 to 15 measurements per dynamic event. Gaussian distribution; Bonferroni’s multiple comparison test; α = 0.05; *P < 0.05, **P < 0.001, ***P < 0.0001, non-indicated pairs show no significance. (**C,F,I**) Schematic representation of RhoA (**C**), Rac1 (**F**) and Cdc42 (**I**) activation patterns related to edge dynamics. All scale bars: 10 μm.

**Figure 5:**
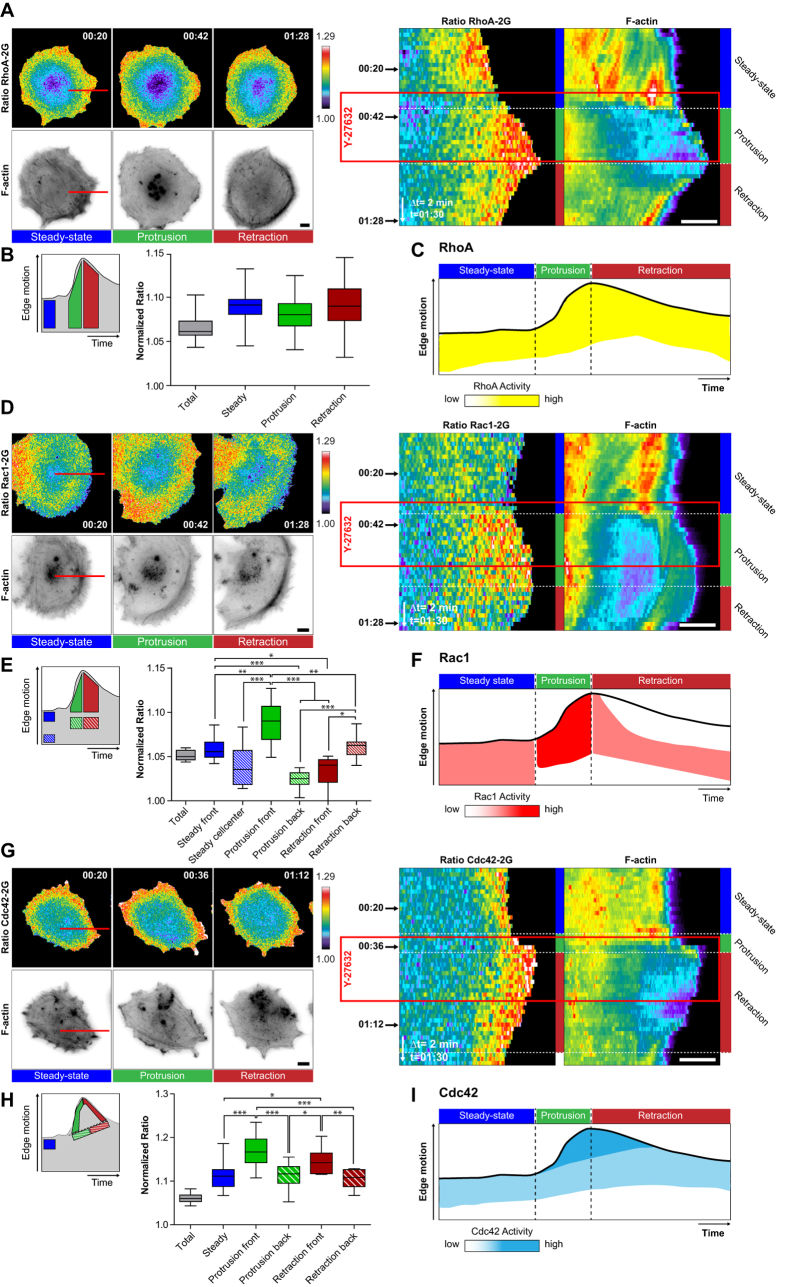
Y-27632 pulse-induced spatio-temporal Rho GTPase activation dynamics. Legend identical to Fig. 4. (**A,D,G**) Spatio-temporal RhoA (**A**), Rac1 (**D**) and Cdc42 (**G**) activation simultaneously measured with F-actin dynamics. (**B,E,H**) Quantification of Rho GTPase signaling states. Total averaged ERs (grey box) were not included in statistical analysis; RhoA: n = 6 cells, 11 to 20 measurements per dynamic event, Rac1: n = 5 cells, 10 to 11 measurements per dynamic event, Cdc42: n = 6 cells 13 to 17 measurements per dynamic event. Non-gaussian distribution, Kruskal-Wallis test followed by Dunn’s multiple comparison post-test; α = 0.05; *P < 0.05, **P < 0.001, ***P < 0.0001. Non-indicated pairs show no significance. (**C,F,I**) Schematic representation of RhoA (**C**), Rac1 (**F**) and Cdc42 (**I**) activation patterns related to edge dynamics.

We then monitored the activity of each Rho GTPase simultaneously with F-actin dynamics, and analyzed Rho GTPase activity fluctuations using kymograph analysis (Fig. 4A). Visual inspection guided us in identifying specific regions of interest (ROIs) within the kymograph, to quantify temporal averages of Rho GTPase activity during specific signaling states (Fig. 4B). Based on these measurements, a schematic representation of Rho GTPase activity fluctuation is presented (Fig. 4C). We also plotted the mean ER value across the whole kymograph, to provide an idea of the relative changes of local versus basal Rho GTPase activity (Figs 4 and 5B,E,H, grey boxplot).

As previously shown^15^, a broad gradient of RhoA activity spanning several micrometers from the edge inwards, occurred at the steady-state (Fig. 4A–C, Movie S5). PDGF-induced protrusion led to rapid RhoA inhibition throughout the cell. During subsequent stalling, RhoA activity then re-appeared at the leading edge. RhoA activity then remained ON during membrane retraction. At steady-state, basal levels of Rac1 activity occurred throughout the cell (Fig. 4D–F, Movie S6). During protrusion, Rac1 activity immediately increased and concentrated in a more focused edge-proximal region, and remained ON during stalling. During retraction, the edge-proximal Rac1 activity zone immediately switched OFF, and a second zone of edge-distal Rac1 activity appeared. These results suggest the existence of two distinct Rac1 activity pools that are differently regulated during protrusion and retraction. At steady-state, Cdc42 was activated in a broad zone at the leading edge (Fig. 4G–I, Movie S7). Cdc42 activity then intensified during PDGF-induced protrusion, stalling and retraction.

**Figure 6:**
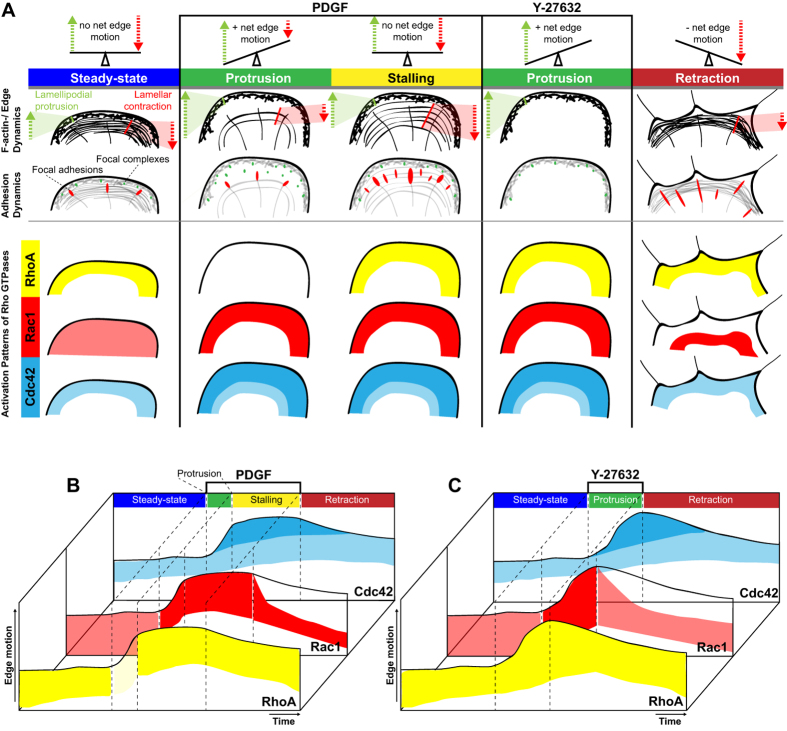
Summary of edge/F-actin and adhesion dynamics in relation to Rho GTPase activation states. (**A**) F-actin and adhesion dynamics are schematically summarized for particular dynamic events (steady state, protrusion, stalling and retraction). First row: Dashed arrows in orientation of motions generated either by the propulsive lamellipodium (green) at the cell periphery or the contractile lamella (red) adjacent to the latter. Adhesion dynamics are displayed in the second row, relating the fate of focal complexes (green) as well as focal adhesions (red) to the respective dynamic state. Below, a scheme summarizes Rho GTPase activation states in a qualitative way, referring to previously depicted edge-/F-actin and adhesion dynamics (RhoA, yellow; Rac1, red; Cdc42,blue). (**B,C**) Observations of spatio-temporal Rho GTPase activation throughout the course of PDGF (**B**) or Y-27632 (**C**) pulse experiments are summarized side by side in schematic kymographs.

Identical measurements in response to Y-27632 revealed a slightly different scenario. Differently from PDGF-triggered protrusions, RhoA activity remained ON throughout the different morphodynamic events (Fig. 5A–C, Movie S8). In contrast, similar Rac1 and Cdc42 activation dynamics as in response to PDGF were observed (Fig. 5D–I, Movies S9,10). Consistently with identical edge morphodynamics as well as F-actin and adhesion dynamics evoked by Y-27632 or PDGF + Y-27632 stimulation, similar spatio-temporal Rho GTPase activation dynamics were observed by both stimulation protocols (Fig. S4). These results indicate the existence of finely regulated spatio-temporal RhoA, Rac1 and Cdc42 signaling programs that correlate with the different F-actin and adhesion dynamics evoked by the different stimuli. A summary of the different edge motility states as well as their associated F-actin, adhesion and Rho GTPase activation dynamics is shown in Fig. 6.

## Discussion

Recent reports have documented complex Rho GTPase signaling programs that regulate leading edge dynamics, and that fluctuate at length and time scales of single microns and tens of seconds^9^,^10^,^11^. With respect to more classic models, this reveals an unanticipated degree of complexity, in which the different Rho GTPases most likely activate different effector pathways in time and space, to fine tune cytoskeletal and adhesion dynamics that enable leading edge motility. Understanding this signaling complexity requires reductionist approaches that allow us to gate on specific morphodynamic events (e.g. leading edge motility versus tail retraction,…). Because these complex signaling programs correlate with rapid and heterogeneous dynamic edge motility states, computer vision and statistical analyses are required to extract the subtle Rho GTPase signaling patterns, and merge them into statistically-relevant datasets^10^,^11^. Here, we use a microfluidic device to induce robust prototypical edge motility behaviors, bypassing cellular heterogeneity to some extent, and allowing us to analyze signaling dynamics using more simple methods. We use our approach to dissect specific Rho GTPase signaling states that are associated with distinct edge motility, F-actin and adhesion dynamics. These prototypical edge motility, and their associated signaling states, operate at a time scale of multiple minutes rather than ten seconds as those observed during fibroblast edge dynamics evoked by simple integrin engagement^10^.

### F-actin and adhesion dynamics correlating with different edge motility states

Our approach reveals stereotypic cytoskeletal and adhesion dynamics associated with the integrin-triggered, poorly motile steady state, and the more robust protrusion and retraction edge motility states induced by PDGF or Y-27632 pulses (Fig. 6A). At steady-state, REF52 cells display robust lamellar actin retrograde flow, most likely in equilibrium with actin polymerization, leading to low net protrusion. This is visible from the robust retrograde flow of radial stress fibers, accompanied by robust focal adhesion maturation. During PDGF-evoked, steady protrusion, the lamellar F-actin network decreases in intensity (as evidenced by a decrease in the intensity of radial stress fibers), and a clear lamellipodial F-actin network is formed. Consistently, this is accompanied with formation of focal complexes that do not mature into focal adhesions. Together, these data strongly suggest decreased myosin-based contractility during PDGF-evoked membrane protrusion. After a couple of minutes, the PDGF-induced edge stalls. We propose that edge geometry/mechanical cues are sensed to activate a mechanical feedback that limits membrane extension, possibly enabling not to overwhelm the limited membrane supply required for protrusion. This correlates with re-enforcement of radial stress fibers, and focal adhesion maturation, indicative of increased myosin-based contractility. However, a robust lamellipodial network can still be observed. We therefore propose that an equilibrium between lamellipodial actin polymerization and lamellar actin retrograde flow leads to PDGF edge stalling. Upon PDGF removal, loss of the protrusive lamellipodial actin network, together with continuous lamellar retrograde actin flow correlates with robust edge retraction.

In contrast to PDGF, Y-27632 triggers very robust edge protrusion without a stalling phase (Fig. 6A). This correlates with formation of robust lamellipodial networks, as well as with an instantaneous loss of lamellar actin retrograde flow, evidently due to inhibition of Rho kinase and myosin-based contractility. This is visible due to disappearance of radial stress fibers, and dissolution of focal adhesions, with only some small remnant adhesions remaining. Compromised mechanosensing due to Y-27632 might also not enable to evoke the stalling phase observed when membrane protrusion is activated using PDGF. Simultaneous stimulation of cells with a pulse of PDGF and Y-27632 evoke identical edge motility, cytoskeletal and adhesions dynamics than Y-27632 alone, indicating that compromised mechanosensing is dominant over chemotropic, PDGF-induced responses (Figs S2 and S4). Not surprisingly, this indicates that mechanosensitive signaling also feeds in the regulation of chemotropic responses. Upon Y-27632 removal, lamellipodial F-actin networks disappear, while lamellar networks re-appear, correlating with edge retraction.

Together, these results suggest that the robust prototypical edge motility states we can induce originate from the interplay of the lamellipodial and lamellar actin networks. Tuning of the strength of both cytoskeletal networks ultimately dictates the edge motility state of the cell.

### Spatio-temporal Rho GTPase activation dynamics associated to prototypical edge motility states

Rather than a universal signaling modality regulating leading egde motility, our results indicate the existence of sophisticated spatio-temporal Rho GTPase signaling networks regulating edge protrusion and retraction. These depend both, on external cues (integrin versus growth factor signaling) and intrinsic cytoskeletal properties (the contractile state of the cell). This might enable to fine tune the cytoskeletal dynamics inherent to different edge motility states. While our approach remains purely correlative, our results suggest that the complex interplay between Rho GTPases might control the balance between lamellipodial actin polymerization versus lamellar actin retrograde flow during edge protrusion, stalling or retraction episodes. Indeed, we can at present only speculate about the possible effector pathways that are switched on in time and space by the different Rho GTPases. A summary of the spatio-temporal Rho GTPase activation dynamics during the different edge dynamics are shown in Fig. 6A,B.

During steady-state, relatively silent edge motility, broad zones of basal Rac1/Cdc42 activities might regulate actin polymerization by activation of Wave/WASP and Arp2/3 proteins^21^, while the broad zone of RhoA activity might control lamellar myosin II-dependent actin through Rho kinases^22^. Consistently, in REF52 cells, lamellar actin retrograde flow and the broad gradient of RhoA activity also correlate with phosphorylated myosin light chain^23^. We propose that the interplay of both signaling activities, and their antagonistic downstream cytoskeletal networks explains the poor net edge protrusion.

During PDGF-evoked membrane protrusion, Rac1 and Cdc42 get activated beyond their steady-state levels, in regions that are more focused at the leading edge, while RhoA activity immediately decreases. Increased (Rac1/Cdc42-dependent) Arp2/3 activation, coupled with decreased (RhoA-dependent) myosin-based retrograde flow might then explain the steady membrane protrusion observed during that phase. These observations suggest that the widely described Rac1/RhoA antagonism^24^ might take place in this specific cellular context. The almost immediate decrease in RhoA activity upon PDGF stimulation suggests the involvement of a rapidly diffusible secondary messenger that might consist of reactive oxygen species, that have previously been described to mediate the Rac1/RhoA antagonism^25^. The finding that Rac1 and RhoA activity zones overlap during steady state protrusion, stalling, and Y-27632-evoked protrusion, suggest that the Rac1/RhoA antagonism is not a universal signaling mechanism, but occurs within specific cellular contexts.

During stalling, Rac1/Cdc42 activity and a lamellipodium remain present. However, a potential mechanosensitive pathway^26^, seems to re-activate RhoA and re-enforce actin retrograde flow to counteract actin polymerization and stall the edge. Edge retraction then correlates with loss of edge-proximal Rac1 activity, and activation of a second, edge-distal, Rac1 pool. RhoA, and surprisingly also Cdc42 remain active. An attractive possibility is that edge-proximal Rac1 inactivation, correlates with loss of Wave/Arp2/3-mediated activation of lamellipodial actin polymerization. In presence of continuous RhoA-mediated actin retrograde flow, edge motility is tipped towards retraction. The significance of continuous Cdc42 activity during retraction is less clear. One scenario is that Cdc42 switches to another effector such as myotonic dystrophy related kinase MRCK, which can co-operate with Rho/Rho kinase signaling to control myosin light chain phosphorylation, and thus contractility^27^. The significance of the appearance of an edge-distal Rac1 activity pool that correlates with retraction is also non-intuitive. However, at this subcellular location, the use of a different effector such as p21 activated kinase (PAK) could feed into the cofilin pathway. Such a spatio-temporal Rac1 signaling network has recently been linked to actin depolymerization during invadosome disassembly^7^.

Our Y-27632 experiments revealed similar spatio-temporal Rac1/Cdc42 activity patterns as for PDGF, showing that these GTPases can sense intracellular variations in contractility, and feed into the regulation of membrane protrusion. RhoA however remained switched ON throughout all morphodynamic phases, even during membrane protrusion. In the latter case, it is important to notice that RhoA won’t contribute to contractile signaling since its effector, Rho kinase, is inhibited. Consistently with previous reports^26^,^28^, these results suggest that compromised mechanosensing in absence of contractility results in an inability to tune RhoA activation dynamics. We show that simultaneous PDGF and Y-27632 pulse lead to identical edge, cytoskeletal, adhesion, and spatio-temporal Rho GTPase signaling dynamics as a simple Y-27632 pulse does. These results underline the importance of mechanosensitive pathways for spatio-temporal control of Rho GTPase activation.

Our results, as well as previous work that has focused on fibroblast edge dynamics that fluctuate on a more rapid timescale^10^ indicate a high degree of complexity of Rho GTPase signaling during fibroblast cell migration. The coming task will now be to characterize the GEFs/GAPs that dynamically shape spatio-temporal patterns of Rho GTPase activity, the mechanisms of the crosstalk between Rho GTPases, and the specific effector pathways that are associated with each Rho GTPase pool to fine tune the different cytoskeletal networks that produce specific edge motility behaviors. Because Rho GTPase activation dynamics fluctuate at second/minute timescales, adequate dissection of this signaling complexity will require optogenetic/chemical biology tools that enable to apply perturbations and manipulate signaling dynamics at these specific timescales.

## Material and methods

### Cdc42 biosensor generation

The Cdc42 FRET biosensor was constructed using the cpFRET toolkit (Fritz *et al.*, 2013). Based on the design of the most sensitive second generation RhoA sensor, we constructed 25 sensor variants consisting of mTFP1 wt and four circular permutations (cps), the CRIB effector domain of WASP (amino acids 201–314; custom gene synthesis; GenScript), a 64 amino acid linker, Venus wt and four cps and Cdc42 itself (custom gene synthesis; GenScript). The design including restriction sites used for cloning as well as the sequence of the second generation Cdc42 (Cdc42-2G) sensor is shown in Fig. S3. This biosensor design preserves the C-terminal lipid modification of Cdc42, which renders the GTPase sensitive to the regulation by RhoGDI. This is a prerequisite to faithfully capture the spatiotemporal activation pattern of endogenous GTPases as observed previously^13^. The most sensitive Cdc42-2G sensor containing mTFP1/cp227 and Venus/cp195 was shuttled from pTriEx into the pAd/CMV/V5-DEST Gateway^®^ Vector according to the manufacturers instructions (Invitrogen). The dominant negative (T17N) and effector (Y40C) mutations as well as the dominant positive (G12V) mutation were generated by site-directed mutagenesis (Stratagene).

### Cdc42 FRET biosensor characterization by fluorometry and microscopy

Analysis of the fluorescence emission spectra of the Cdc42 biosensor library and Cdc42-2G dominant positive and negative mutants were performed on a fluorometer (Perkin Elmer LS50b). Typically, 2 × 10^5^ HEK293FT cells were seeded into a 12-well cell culture plate and transfected with 100 ng of biosensor, 400 ng of RhoGDI or GAPs and 1000 ng of GEFs using Metafectene (Biontex). After 48 hours, cells were detached with brief trypsin treatment and resuspended in ice-cold PBS. Cell suspensions were measured in a quartz cuvette. Cdc42 sensor was excited at 460 nm and emission spectra were recorded from 480–600 nm with a step of 0.5 nm. Spectra were background-subtracted with the spectra of non-transfected cells and normalized according to their spectrum integral. Relative FRET was calculated by dividing the normalized fluorescence intensity of Venus at its emission peak (528 nm) by the normalized fluorescence intensity of mTFP1 at its emission peak (492 nm). The response of the Cdc42-2G sensor to GEFs and GAPs was analyzed by microscopy. HEK293FT cells were seeded into 96-well plate (Falcon) coated with 10 μg/ml Poly-L-lysine and transfected as described above. 9 fields of view were acquired per experiment using a Plan Apo 10x objective and the Screening Acquisition Module in MetaMorph (Molecular Devices) and the average ratio per field of view was than calculated from ≥100 cells.

### Cell culture, generation of stable cell lines and infection with adenoviral vectors

Rat embryonic fibroblasts (REF52) were cultured in Dulbecco’s modified eagle medium (DMEM; Sigma) supplemented with 10% fetal bovine serum (FBS; Sigma), 100 U/ml penicillin/ streptomycin (Sigma) and 4 mM L-Glutamine (Sigma). For serum starvation and imaging, Ham’s Nutrient Mixture F12 (Ham’s F12, Sigma), supplemented with 0.5% FBS and 0.5% bovine serum albumin (BSA; Sigma), 100 U/ml penicillin/ streptomycin, 4 mM L-Glutamine was used.

Stable cell lines expressing VASP-GFP, or the RhoA FRET biosensor RhoA2G were described previously^15^. Similarly, stable cell lines expressing Paxillin-mCherry were constructed. For simultaneous imaging of F-actin dynamics, these stable cells were infected with an adenovirus encoding Lifeact-mCherry^15^. Long term expression of Rac1-2G^19^, and Cdc42-2G (this study) using lentiviral transduction led to obvious cell morphological aberrations. This problem was solved by short-term biosensor expression using adenoviral vectors. In these experiments, double infections with adenoviruses encoding for Rac1-2G or Cdc42-2G, and Lifeact-mCherry were performed. Adenoviral particles from crude packaging cell lysates were frozen as aliquots. After titration, these crude lysates were used at appropriate concentration for cell infection. REF52 cells were infected for 16 hours, and switched to complete medium for 24 hours. Cells were then seeded in microfluidic devices in starving medium, and imaging started one hour post-seeding.

### Construction and handling of microfluidic devices

We used a modified version of a previously-described microfluidic circuit to apply chemokine gradient to cells^29^. Slight modifications of the construction of the microfluidic circuit allowed us to deliver growth factor pulses. The microfluidic silicon master was replicated from a Silicon wafer with SU-8 micro-structures. The silicon master mold was composed of two 40 μm and 100 μm thickness layers of photoresist. First, the plasma treated Silicon wafer was spin-coated with SU-8 100 (Microchem, USA) negative thick photoresist until a height of 40 μm was achieved. After baking at 65 °C for 5′ and 95 °C for 20′, the wafer was exposed to 405 nm ultraviolet light (Shinu MST, Korea) with a 250 mJ dose and masked by the negative film mask (Han&All Tech, Korea). After this first round of exposure, the wafer was baked again at 65 °C for 1′ and 95 °C for 10′. SU-8 developer (Microchem, USA) was then used to remove the unexposed part of the photoresist. For deposition of the second photoresist layer, the film mask for the second master was correctly positioned using the alignment pattern on the first layer of the wafer. The photoresist for the second layer was then spin-coated until a 100 μm thickness was achieved. The wafer was baked at 65 °C for 10′ and 95 °C for 30′, and exposed to 500 mJ of 405 nm UV light. After a final baking step at 65 °C for 1′ and 95 °C for 10′, the wafer was dipped into the developer, and baked to evaporate the residual solvents on the top.

Polydimethylsiloxane (PDMS) was used to replicate the master. The precursor (Sylgard 184, Dow Corning) was mixed at a 10:1 ratio and degassed in a vacuum chamber for 5′. 7 g precursor was then poured on the top of the master, and solidified at 80 °C in a dry oven for 30′. The plastic reservoir from a 8-well strip (Evergreen sci, USA) was then glued on top of the microfluidic device using precursor. These reservoirs contain the medium and growth factors, and allow us to connect the microfluidic device to the ONIX pressure pump (Millipore). An additional layer of 30 gr. of precursor was added to seal the plastic reservoir. As shown in Supplementary Information Fig. 1A, the PDMS replica was cut and punched. Glass coverslips (50 × 70 mm) were thoroughly washed with anhydrous ethanol and demineralized water subsequently. PDMS devices were placed bottom side up together with dried coverslips into a CUTE plasma oven (Femto Science) and plasma treated at 100% power for 1′. After plasma treatment, the PDMS device was mounted on the glass coverslip and further incubated for 15′ at 70 °C to enhance efficiency of covalent bonding. Devices were then coated with 20 μg/ml fibronectin, and stored over night at 4 °C prior to imaging experiments.

### Live cell imaging

One hour prior to imaging, cells were reseeded subconfluently on fibronectin-coated microfluidic devices under serum starving conditions using Ham’s F12 starvation medium. Tubing connected to a pressure pump (ONIX, Millipore) was plugged to the inlets of the device and outlet reservoirs were covered with Parafilm to minimize medium evaporation.

One out of the two inlets of each device was filled with Ham’s F12 medium alone, while the other was loaded with Ham’s F12 supplemented with 4 μM 10 kDa Dextran-Alexa 647 (Invitrogen), 40 ng/ml of PDGF or 10 μg/ml Y27632, respectively. Immediately after, pressure of 0.5 psi on the reservoir was applied on the inlet containing only medium to prevent diffusion of PDGF or Y27632 into the cell chamber of the microfluidic device. During the PDGF/Y-27632 pulse experiments, medium exchanges were performed applying 1 psi for two minutes and further pressure was reduced to 0.5 psi for medium maintenance.

All imaging experiments were performed on an Eclipse Ti inverted fluorescence microscope (Nikon) with Plan Apo VC λ Oil 60× (NA 1.4), Apo TIRF 60× Oil (NA 1.49) or Plan Apo Lambda DM Air 40× (NA 0.95) objectives controlled by MetaMorph (Molecular Devices) software. Laser-based autofocus was used throughout the experiments. A CoolLED lamp with 440 nm (mTeal excitation), or GYR LEDs (mCherry or Alexa647 excitation) were used as light sources with appropriate excitation filters. FRET experiments were performed as described elsewhere^13^. For TIRF imaging, 491 or 561 nm solid-state laser diodes integrated within a TIRF illumination system were used (Roper Scientific). FRET/mCherry illumination experiments were acquired using a Hamamatsu Orca R2 CCD camera. GFP/mCherry TIRF imaging experiments were acquired using a Photometrics Evolve EMCCD. All images were acquired at 16-bit depth.

### Image Analysis

Basic image analysis and processing was performed using MetaMorph software. FRET data were analyzed as described elsewhere^13^. Edge velocities were assessed using the ImageJ plugin ADAPT^30^. Adhesion intensities at distinct dynamic events were analyzed employing the Focal Adhesion Analysis Server (FAAS)^31^.

## Additional Information

**How to cite this article**: Martin, K. *et al.* Spatio-temporal co-ordination of RhoA, Rac1 and Cdc42 activation during prototypical edge protrusion and retraction dynamics. *Sci. Rep.* **6**, 21901; doi: 10.1038/srep21901 (2016).

## Supplementary Material

Supplementary Information

Supplementary Movie S1

Supplementary Movie S2

Supplementary Movie S3

Supplementary Movie S4

Supplementary Movie S5

Supplementary Movie S6

Supplementary Movie S7

Supplementary Movie S8

Supplementary Movie S9

Supplementary Movie S10
