# Supplementary material for: Spatio-temporal co-ordination of RhoA, Rac1 and Cdc42 activation during prototypical edge protrusion and retraction dynamics
Source: Sci Rep. 2016 Feb 25;6:21901. doi: 10.1038/srep21901 (PMC4766498; doi:10.1038/srep21901)
Supplement: Supplementary Information [file srep21901-s1.pdf]

Supplementary Information

Spatio-temporal co-ordination of RhoA, Rac1 and Cdc42 activation during prototypical edge protrusion and retraction dynamics

Katrin Martin [1], Andreas Reimann [1,2], Rafael D. Fritz [1], Hyunryul Ryu [3,4], Noo Li Jeon [3,4], Olivier Pertz [1,5*]

[1]Dept. of Biomedicine, University of Basel, Mattenstrasse 28, 4058 Basel, Switzerland

[2] Present address: Department of Biosystems, Science and Engineering (D-BSSE), ETH Zurich, Mattenstrasse 26, 4058 Basel, Switzerland

[3]School of Mechanical and Aerospace Engineering, Seoul National University, Seoul, 151-742, Republic of Korea

[4]Institute of Advanced Machinery and Design, Seoul National University, Seoul, 151-742, Republic of Korea

[5] Present address: Institute of Cell Biology, University of Bern, Baltzerstrasse 4, 3012 Bern, Switzerland

*Correspondence and requests for materials should be addressed to O.P. (email: olivier.pertz@izb.unibe.ch)

Supplementary Information

Supplementary Figures

Figure S1. Microfluidic control of pulsed GF/drug delivery.
(A) Scheme of microfluidic setup. The microfluidic device consists of a control part with different inlets connected via resistances to a cell chamber and finally to a sink. A computer-controlled pressure pump facilitates medium exchanges at low flow rates.
(B) A fluorescently-labeled 10 kDa dextran was co-imaged in every experiment as a marker for fidelity of growth factor/ drug pulse delivery. A representative kymograph and micrograph are shown as examples for visualization of the growth factor/ drug pulse.
(C, D) Effects of flow on edge dynamics. (C) Representative micrographs of F-actin signals (visualized using Lifeact-mCherry) in presence (top panels) or absence (bottom panels) of PDGF stimulation. Top box represents fluorescently-labeled dextran. (D) Kymographs of F-actin (Lifeact) and fluorescently-labeled 10 kDa dextran signal corresponding to cells in (C). Kymographs follow black line drawn in micrographs of (C). Red outline marks dextran pulses with and without PDGF, respectively. Note lack of membrane protrusion in absence of PDGF stimulation.
Scale bars: (B - D) 50 μm

Figure S2. PDGF + Y-27632 pulse-induced edge/ F-actin and adhesion dynamics.
(A) Quantification of edge dynamics upon PDGF + Y-27632 stimulation. The ADAPT Image J plugin [1] was used to extract edge dynamics and velocities using the Lifeact-mCherry signal.
Left panel: cell outlines display color-coded protrusion/ retraction velocities (Time scale: hours:minutes). Right panel: velocity maps along the entire cell edge at normalized positions. Black dashed contours indicate specific cell outlines shown in the left panel. Color-code according to the scale bar.

(B) Cell area dynamics in response to PDGF/ Y-27632/ PDGF + Y-27632. Cell area was measured using ADAPT, and normalized to t = 0'. Average area ± s.e.m (n=10 cells).

(C) Quantification of F-actin fluctuations. Whole cell Lifeact-mCherry fluorescence intensity was averaged in response to a PDGF/ Y-27632/ PDGF + Y-27632 pulse. (n = 5 cells)

(D) F-actin dynamics during protrusion/ retraction in response to PDGF + Y-27632. F-actin signals are color-coded for signal intensity or shown in inverted black and white (ibw) contrast.

Left panel: F-actin signal (color-coded) of representative whole cells (left), as well as magnified insets (successively corresponding to the white and black dashed boxes). White solid lines represent ROIs used for kymograph analysis.

Right panel: Kymograph analysis of F-actin dynamics during the PDGF + Y-27632 pulse.

(E) Representative adhesion dynamics in response to a PDGF + Y-27632 pulse. Cells expressing Paxillin-mCherry were imaged using TIRF microscopy. Kymograph analysis of adhesion dynamics in ibw contrast. Magnifications of selected insets from the kymograph (depicted by color-coded boxes corresponding to prototypical morphodynamic states) are also shown. In the insets, a dashed crosshair provides a virtual reference for visual inspection of the motile behavior of the adhesions. Note that all the images have been scaled identically for a fair comparison of fluorescence intensities.

(F) Quantification of adhesion fluorescence intensity in response to a PDGF + Y27632 pulse. Single adhesions were segmented using the Focal Adhesion Analysis Server (FAAS) [2]. Average fluorescence intensities were computed and normalized to the pre-pulse, steady-state. Boxplots with median, interquartile (box) and 1.5 IQR (whiskers) range with the fluorescence intensity distributions are shown; n = 6 cells, 2200-2400 adhesions per condition; Non-gaussian distribution, Kruskal-Wallis test followed by Dunn's multiple comparison post-test.; $\alpha = 0.05$; ***$p < 0.0001$.

Scale bars: (A, D) 10 µm. (E) 5 µm

Figure S3. Additional characterization of the Cdc42 biosensor library.

(A) Emission profiles of the 25 Cdc42 biosensor variants. HEK293FT cells transfected with the indicated biosensors were excited at 460 nm and emission spectra recorded from 480 to 600 nm in a fluorometer. Normalized emission profiles are shown in the ON (black trace) and OFF (red trace) states. Spectra are normalized by the area under the curve. $\Delta R/R_0$ values of the indicated biosensors are shown in percentage.

(B) Sequence of Cdc42-2G (mTFP1/cp227-Venus/cp195) biosensor. Different biosensor domains are color-coded according to the scheme displayed right to the sequence. Different expression and viral vectors plasmids are available at Addgene. Plasmid ID: 68814 (pTriEx-Cdc42-2G), 68813 (pLenti-Cdc42-2G), 68812 (pAd/CMV/V5-DEST-Cdc42-2G).

(C) Emission profiles of the indicated Cdc42 biosensor mutants compared to the wild type (WT) in their ON states.

Figure 4. PDGF + Y-27632 pulse-induced spatio-temporal Rho GTPase activation dynamics.

(A, D, G) Spatio-temporal RhoA (A), Rac1 (D) and Cdc42 (G) activation simultaneously measured with F-actin dynamics. Left panel: Rho GTPase activation and F-actin signals representative of prototypical morphodynamic states. ERs are color-coded (top), and F-actin signals are shown in ibw contrast (bottom). Right panel: Kymograph analysis of ERs, and F-actin (color-coded for fluorescence intensity) along the red line shown in the micrographs. Time scale: hours:minutes.

(B, E, H) Quantification of Rho GTPase signaling states. Left panel: schematic representation of kymograph ROIs that were used to quantify Rho GTPase activity. Right panel: Boxplots of ROI-averaged ERs, with median, interquartile (box) and 1.5 IQR (whiskers). ERs were normalized by the average of the 10% pixels with lowest ERs, representing basal Rho GTPase activity within the cell, enabling to compare ERs across different biosensors. RhoA: n = 5 cells, 12 to 13 measurements per dynamic event, Rac1: n = 5 cells, 10 to 11 measurements per dynamic event. Non-gaussian distribution, Kruskal-Wallis test followed by Dunn's multiple comparison post-test; $\alpha = 0.05$; * $P<0.05$, **

P<0.001, *** P<0.0001, non-indicated pairs show no significance; Cdc42: n = 4 cells 12 measurements per dynamic event. Gaussian distribution; Bonferroni's multiple comparison test; α = 0.05; * P<0.05, ** P<0.001, *** P<0.0001

(C, F, I) Schematic representation of RhoA (C), Rac1 (F) and Cdc42 (I) activation patterns related to edge dynamics.

All scale bars: 10 μm.

References:

1 Barry, D. J., Durkin, C. H., Abella, J. V. & Way, M. Open source software for quantification of cell migration, protrusions, and fluorescence intensities. *The Journal of cell biology* **209**, 163-180 (2015).

2 Berginski, M. E. & Gomez, S. M. The Focal Adhesion Analysis Server: a web tool for analyzing focal adhesion dynamics. *F1000Research* **2** (2013).

Supplementary Figure S1

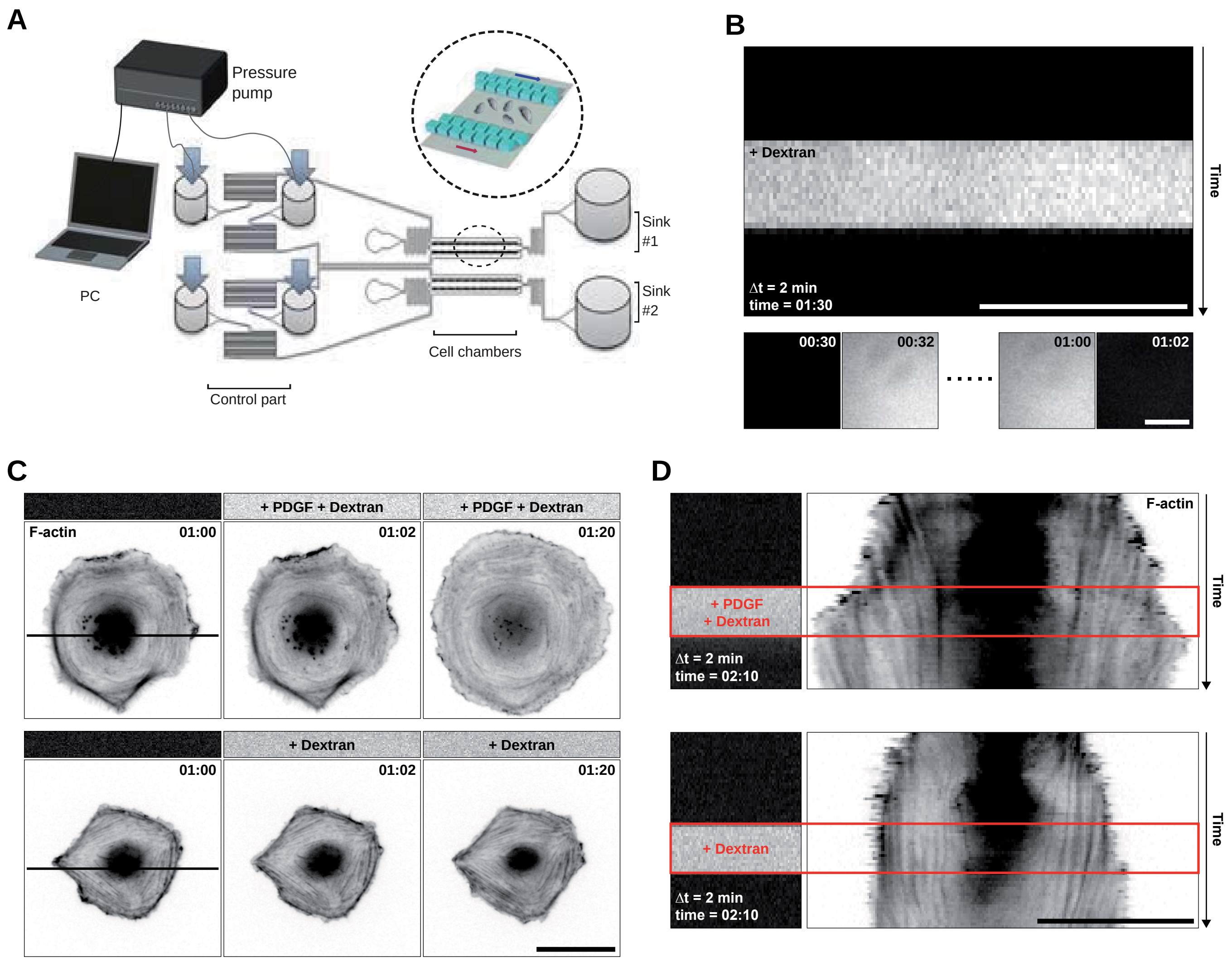

Supplementary Figure S2

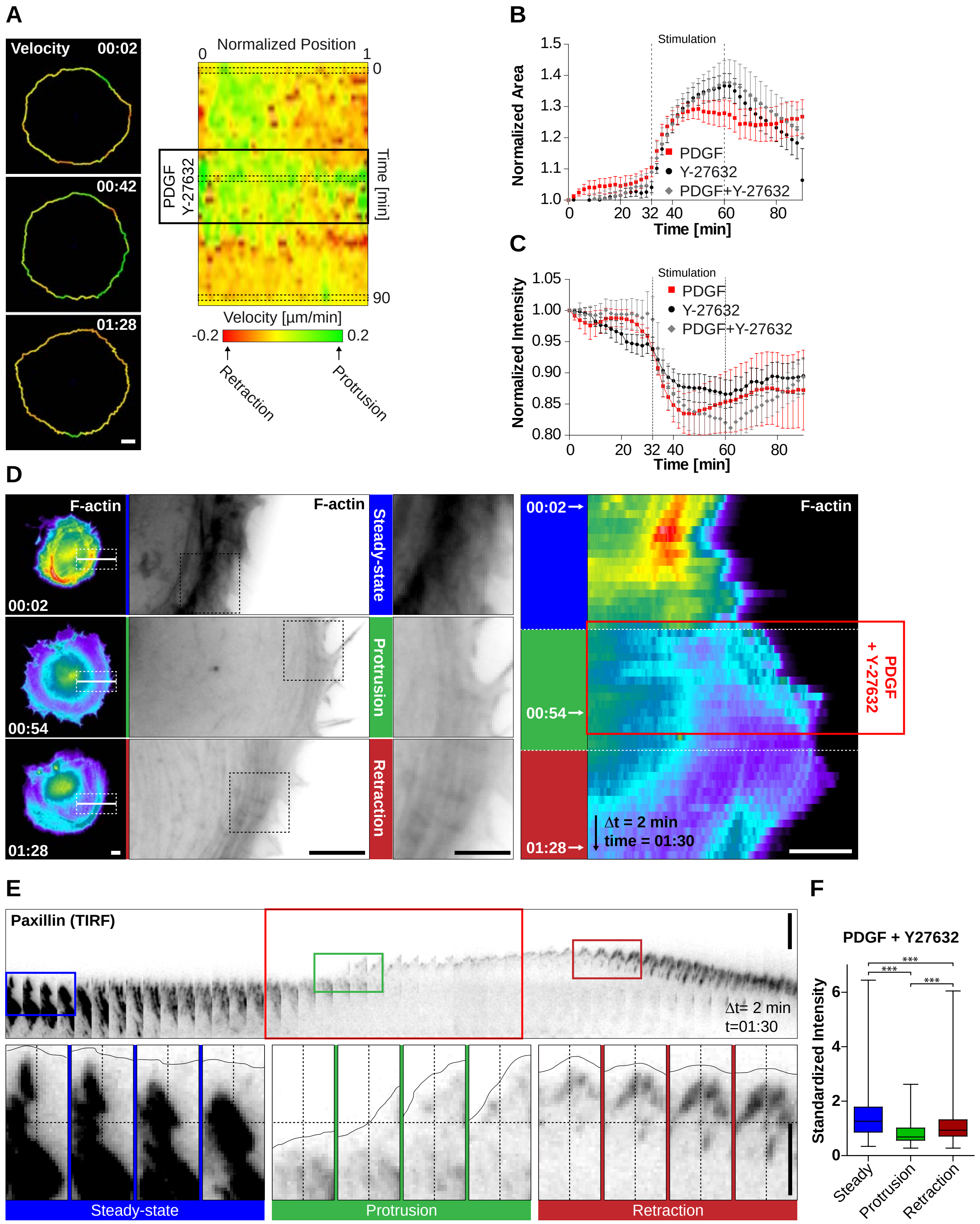

Supplementary Figure S3

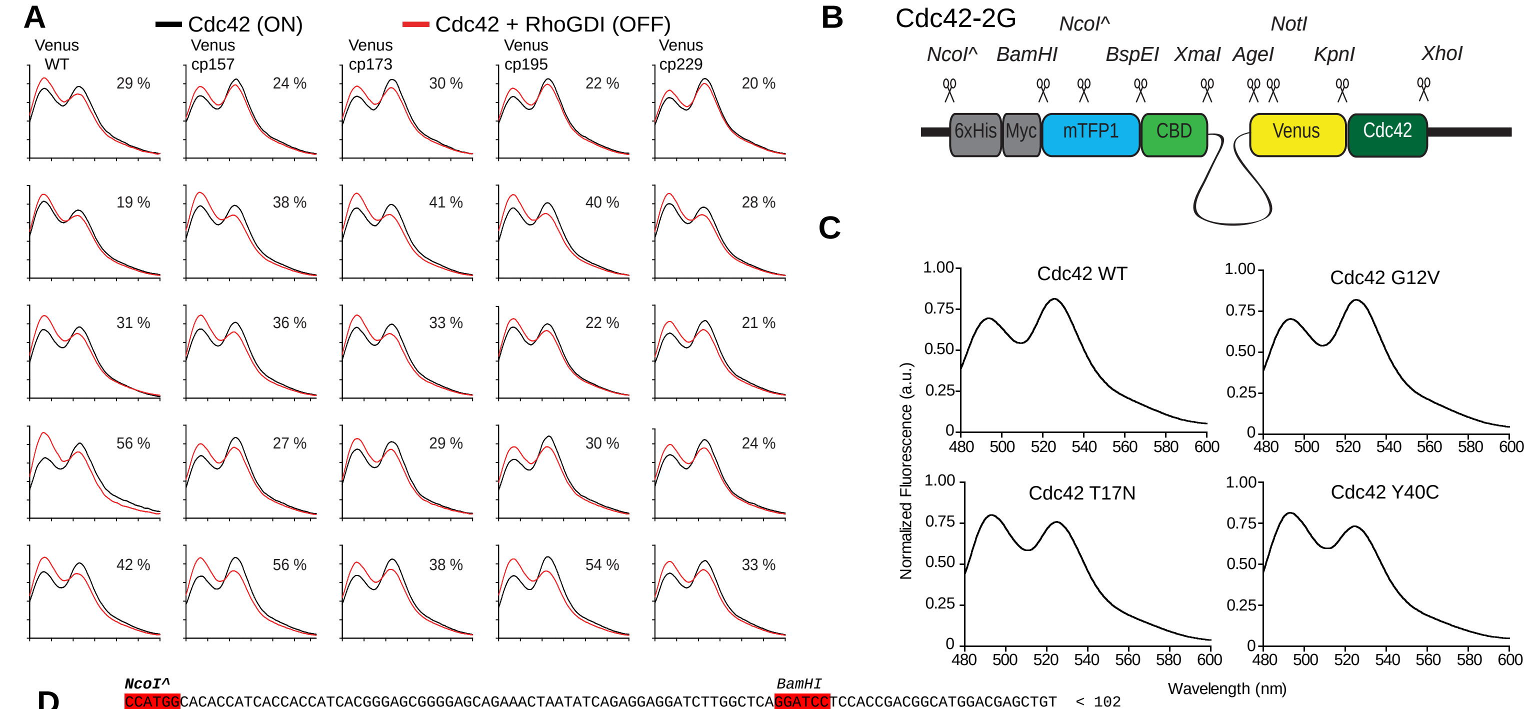

D

```
NcoI^                                                                        BamHI
CCATGGCACACCATCACCACCATCACGGGAGCGGGGAGCAGAAACTAATATCAGAGGAGGATCTTGGCTCAGGATCCTCCACCGACGGCATGGACGAGCTGT  < 102
  M  A  H  H  H  H  H  H  G  S  G  E  Q  K  L  I  S  E  E  D  L  G  S  G  S  S  T  D  G  M  D  E  L  Y

ACAAGGGTGCTAGCGGAGGAATGGTGAGCAAGGGCGAGGAGACCACAATGGGCGTAATCAAGCCCGACATGAAGATCAAGCTGAAGATGGAGGGCAACGTGA  < 204
  K  G  A  S  G  G  M  V  S  K  G  E  E  T  T  M  G  V  I  K  P  D  M  K  I  K  L  K  M  E  G  N  V  N

ATGGCCACGCCTTCGTGATCGAGGGCGAGGGCGAGGGCAAGCCCTACGACGGCACCAACACCATCAACCTGGAGGTGAAGGAGGGAGCCCCCCTGCCCTTCT  < 306
  G  H  A  F  V  I  E  G  E  G  E  G  K  P  Y  D  G  T  N  T  I  N  L  E  V  K  E  G  A  P  L  P  F  S

CCTACGACATTCTGACCACCGCGTTCGCCTACGGCAACAGGGCCTTCACCAAGTACCCCGACGACATCCCCAACTACTTCAAGCAGTCCTTCCCCGAGGGCT  < 408
  Y  D  I  L  T  T  A  F  A  Y  G  N  R  A  F  T  K  Y  P  D  D  I  P  N  Y  F  K  Q  S  F  P  E  G  Y

                                                                      NcoI^
ACTCTTGGGAGCGCACCATGACCTTCGAGGACAAGGGCATCGTGAAGGTGAAGTCCGACATCTCCATGGAGGAGGACTCCTTCATCTACGAGATACACCTCA  < 510
  S  W  E  R  T  M  T  F  E  D  K  G  I  V  K  V  K  S  D  I  S  M  E  E  D  S  F  I  Y  E  I  H  L  K

AGGGCGAGAACTTCCCCCCCAACGGCCCCGTGATGCAGAAGAAGACCACCGGCTGGGACGCCTCCACCGAGAGGATGTACGTGCGCGACGGCGTGCTGAAGG  < 612
  G  E  N  F  P  P  N  G  P  V  M  Q  K  K  T  T  G  W  D  A  S  T  E  R  M  Y  V  R  D  G  V  L  K  G

GCGACGTCAAGCACAAGCTGCTGCTGGAGGGCGGCGGCCACCACCGCGTTGACTTCAAGACCATCTACAGGGCCAAGAAGGCGGTGAAGCTGCCCGACTATC  < 714
  D  V  K  H  K  L  L  L  E  G  G  G  H  H  R  V  D  F  K  T  I  Y  R  A  K  K  A  V  K  L  P  D  Y  H

                                                                                          BspEI
ACTTTGTGGACCACCGCATCGAGATCCTGAACCACGACAAGGACTACAACAAGGTGACCGTTTACGAGAGCGCCGTGGCCCGCAACTCCGGAGACATCCAGA  < 816
  F  V  D  H  R  I  E  I  L  N  H  D  K  D  Y  N  K  V  T  V  Y  E  S  A  V  A  R  N  S  G  D  I  Q  N

ACCCTGACATCACGAGTTCACGATACCGTGGGCTCCCAGCACCTGGACCTAGCCCAGCTGATAAGAAACGCTCAGGGAAGAAGAAGATCAGCAAAGCTGATA  < 918
  P  D  I  T  S  S  R  Y  R  G  L  P  A  P  G  P  S  P  A  D  K  K  R  S  G  K  K  K  I  S  K  A  D  I

TTGGTGCACCCAGTGGATTCAAGCATGTCAGCCACGTGGGGTGGGACCCCCAGAATGGATTTGACGTGAACAACCTCGACCCCGACCTGCGCAGTCTGTTCT  < 1020
  G  A  P  S  G  F  K  H  V  S  H  V  G  W  D  P  Q  N  G  F  D  V  N  N  L  D  P  D  L  R  S  L  F  S

CCAGGGCAGGAATCAGCGAGGCCCAGCTCACCGACGCCGAGACCTCTAAACTTATCTACGACTTCATTGAGGACCAGGGTGGGCTGGAGGCTGTGCGGCAGG  < 1122
  R  A  G  I  S  E  A  Q  L  T  D  A  E  T  S  K  L  I  Y  D  F  I  E  D  Q  G  G  L  E  A  V  R  Q  E

                          XmaI
AGATGAGGCGCCAGGAGCCACTTCCGCCCGGGACTTCTGGTTCTGGTAAACCTGGTTCTGGTGAAGGTTCTACTAAAGGGACTTCTGGTTCTGGTAAACCTG  < 1224
  M  R  R  Q  E  P  L  P  P  G  T  S  G  S  G  K  P  G  S  G  E  G  S  T  K  G  T  S  G  S  G  K  P  G

GTTCTGGTGAAGGTTCTACTAAAGGGACTTCTGGTTCTGGTAAACCTGGTTCTGGTGAAGGTTCTACTAAAGGGACTTCTGGTTCTGGTAAACCTGGTTCTG  < 1326
  S  G  E  G  S  T  K  G  T  S  G  S  G  K  P  G  S  G  E  G  S  T  K  G  T  S  G  S  G  K  P  G  S  G

                    NotI
GTGAAGGTTCTACTAAAGGGGCGGCCGCCATGCTGCTGCCCGACAACCACTACCTGAGCTACCAGTCCGCCCTGAGCAAAGACCCCAACGAGAAGCGCGATC  < 1428
  E  G  S  T  K  G  A  A  A  M  L  L  P  D  N  H  Y  L  S  Y  Q  S  A  L  S  K  D  P  N  E  K  R  D  H

ACATGGTCCTGCTGGAGTTCGTGACCGCCGCCGGGATCACTCTCGGCATGGACGAGCTGTACAAGGGTGGCAGCGGTGGCATGGTGAGCAAGGGCGAGGAGC  < 1530
  M  V  L  L  E  F  V  T  A  A  G  I  T  L  G  M  D  E  L  Y  K  G  G  S  G  G  M  V  S  K  G  E  E  L

TGTTCACCGGGGTGGTGCCCATCCTGGTCGAGCTGGACGGCGACGTAAACGGCCACAAGTTCAGCGTGTCCGGCGAGGGCGAGGGCGATGCCACCTACGGCA  < 1632
  F  T  G  V  V  P  I  L  V  E  L  D  G  D  V  N  G  H  K  F  S  V  S  G  E  G  E  G  D  A  T  Y  G  K

AGCTGACCCTGAAGCTGATCTGCACCACCGGCAAGCTGCCCGTGCCCTGGCCCACCCTCGTGACCACCCTGGGCTACGGCCTGATGTGCTTCGCCCGCTACC  < 1734
  L  T  L  K  L  I  C  T  T  G  K  L  P  V  P  W  P  T  L  V  T  T  L  G  Y  G  L  M  C  F  A  R  Y  P

CCGACCACATGAAGCAGCACGACTTCTTCAAGTCCGCCATGCCCGAAGGCTACGTCCAGGAGCGCACCATCTTCTTCAAGGACGACGGCAACTACAAGACCC  < 1836
  D  H  M  K  Q  H  D  F  F  K  S  A  M  P  E  G  Y  V  Q  E  R  T  I  F  F  K  D  D  G  N  Y  K  T  R

GCGCCGAGGTGAAGTTCGAGGGCGACACCCTGGTGAACCGCATCGAGCTGAAGGGCATCGACTTCAAGGAGGACGGCAACATCCTGGGGCACAAGCTGGAGT  < 1938
  A  E  V  K  F  E  G  D  T  L  V  N  R  I  E  L  K  G  I  D  F  K  E  D  G  N  I  L  G  H  K  L  E  Y

ACAACTACAACAGCCACAACGTCTATATCACCGCCGACAAGCAGAAGAACGGCATCAAGGCCAACTTCAAGATCCGCCACAACATCGAGGACGGCGGCGTGC  < 2040
  N  Y  N  S  H  N  V  Y  I  T  A  D  K  Q  K  N  G  I  K  A  N  F  K  I  R  H  N  I  E  D  G  G  V  Q

                                                   KpnI
AGCTCGCCGACCACTACCAGCAGAACACCCCCATCGGCGACGGCCCCGTGGGTACCATGCAGACAATTAAGTGTGTTGTTGTTGGTGATGGTGCTGTTGGTA  < 2142
  L  A  D  H  Y  Q  Q  N  T  P  I  G  D  G  P  V  G  T  M  Q  T  I  K  C  V  V  V  G  D  G  A  V  G  K

AAACATGTCTCCTGATATCCTACACAACAAACAAATTCCCATCGGAATATGTACCAACTGTTTTTGACAACTATGCAGTCACAGTTATGATTGGTGGAGAGC  < 2244
  T  C  L  L  I  S  Y  T  T  N  K  F  P  S  E  Y  V  P  T  V  F  D  N  Y  A  V  T  V  M  I  G  G  E  P

CATACACTCTTGGACTTTTTGATACTGCAGGGCAAGAGGATTATGACAGACTACGACCGCTAAGTTATCCACAGACAGATGTTTTTCTAGTATGTTTCTCAG  < 2346
  Y  T  L  G  L  F  D  T  A  G  Q  E  D  Y  D  R  L  R  P  L  S  Y  P  Q  T  D  V  F  L  V  C  F  S  V

TGGTCTCTCCATCCTCATTTGAAAATGTGAAAGAAAAGTGGGTGCCTGAGATAACTCACCACTGTCCAAAGACTCCTTTCTTGCTTGTTGGGACCCAAATTG  < 2448
  V  S  P  S  S  F  E  N  V  K  E  K  W  V  P  E  I  T  H  H  C  P  K  T  P  F  L  L  V  G  T  Q  I  D

ATCTCAGAGATGACCCCTCTACTATTGAGAAACTTGCCAAGAACAAACAGAAGCCTATTACTCCAGAGACTGCTGAAAAGCTGGCGCGGGATCTGAAGGCTG  < 2550
  L  R  D  D  P  S  T  I  E  K  L  A  K  N  K  Q  K  P  I  T  P  E  T  A  E  K  L  A  R  D  L  K  A  V

TCAAGTATGTGGAGTGCTCTGCCCTCACACAGAAAGGCCTAAAGAATGTGTTTGATGAAGCAATATTGGCTGCCCTGGAGCCTCCAGAACCGAAGAAGAGCC  < 2652
  K  Y  V  E  C  S  A  L  T  Q  K  G  L  K  N  V  F  D  E  A  I  L  A  A  L  E  P  P  E  P  K  K  S  R

                 XholI
GCAGGTGTGTGCTGCTATGACTCGAG  < 2678      ^ = double cutter
  R  C  V  L  L  *  L  E
```

Supplementary Figure S4

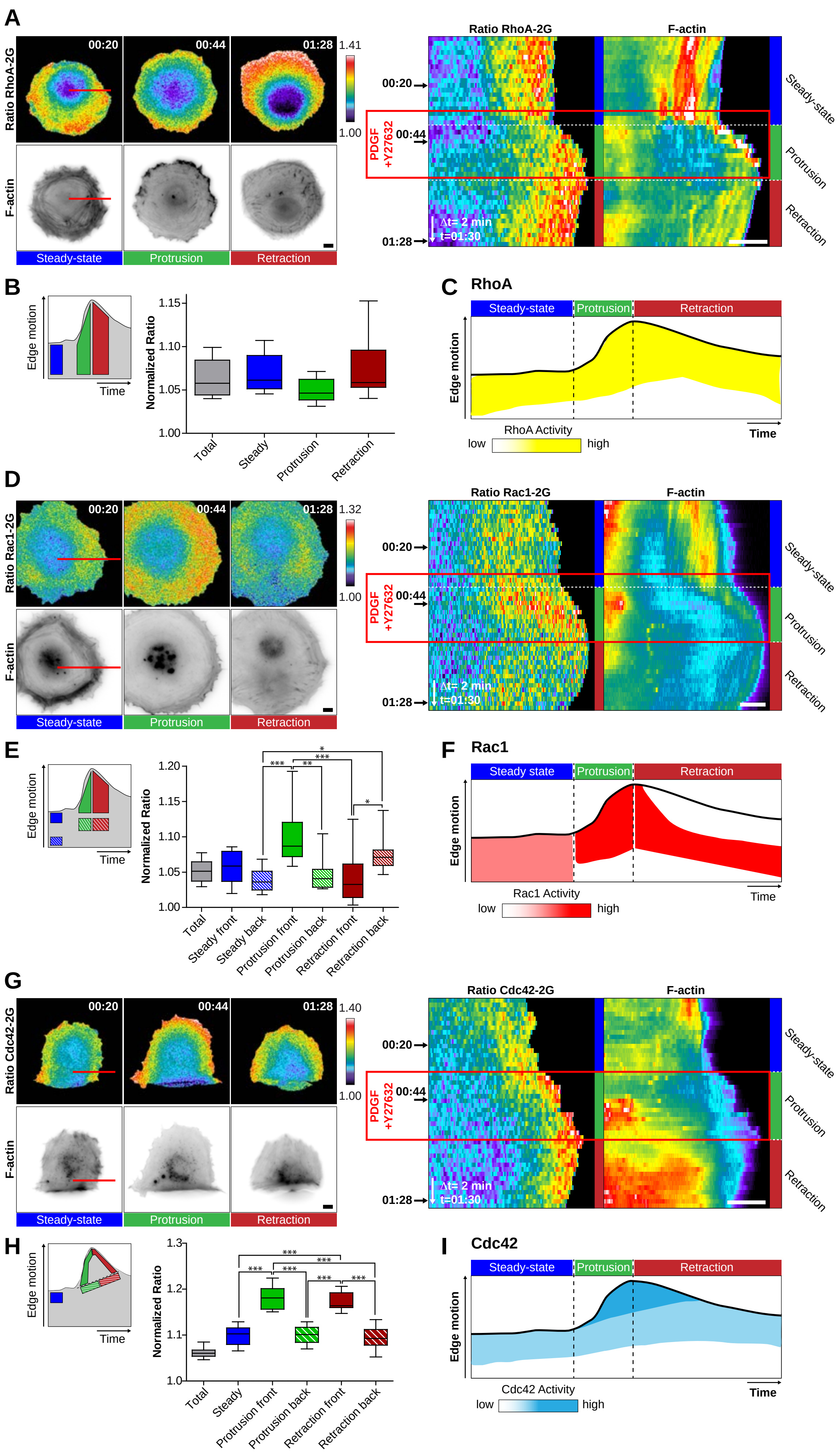

Supplementary Videos

Video 1 - Edge/F-actin dynamics in response to a PDGF pulse (Related to Fig.1)

A REF52 cell expressing Lifeact-mCherry was imaged using epifluorescence microscopy. Upper left panel: Color-coded cell outline according to edge velocity (Fig.1A). Upper right panel: F-actin channel color-coded according to fluorescence intensity. Lower panel: magnification of the F-actin dynamics in the inset (ibw contrast). Red bar indicates PDGF pulse. Time scale: hours: minutes. Scale bars: 10 μm.

Video 2 - Edge/F-actin dynamics in response to a Y-27632 pulse (Related to Fig.1)

A REF52 cell expressing Lifeact-mCherry was imaged using epifluorescence microscopy. Upper left panel: Color-coded cell outline according to edge velocity (Fig.1B). Upper right panel: F-actin channel color-coded according to fluorescence intensity. Lower panel: magnification of the F-actin dynamics in the inset (ibw contrast). Red bar indicates Y-27632 pulse. Time scale: hours: minutes. Scale bars: 10 μm.

Video 3 - Adhesion and F-actin dynamics in response to a PDGF pulse (Related to Fig. 2)

A REF52 cell expressing Lifeact-mCherry (F-actin) and VASP-GFP (adhesion) was imaged using epifluorescence microscopy (Lifeact), and TIRF (VASP) microscopy. Left panel: F-actin signal in ibw contrast. Right panel: VASP-GFP signal in the magnified inset color-coded for fluorescence intensity. Red bar indicates PDGF pulse. Time scale: hours: minutes. Scale bars: 10 μm.

Video 4 - Adhesion and F-actin dynamics in response to a Y-27632 pulse (Related to Fig. 2)

A REF52 cell expressing Lifeact-mCherry (F-actin) and VASP-GFP (adhesion) was imaged using epifluorescence microscopy (Lifeact), and TIRF (VASP) microscopy. Left panel: F-actin signal in ibw contrast. Right panel: VASP-GFP signal in the magnified inset color-coded for fluorescence intensity. Red bar indicates Y-27632 pulse. Time scale: hours: minutes. Scale bars: 10 μm.

Video 5 - RhoA activation and F-actin dynamics in response to a PDGF pulse

(Related to Fig. 4)

A REF52 cell expressing Lifeact-mCherry (F-actin) and the RhoA-2G biosensor was imaged using epifluorescence microscopy. Left panel: ERs are shown in color code (dynamic range: 1 - 1.24). Right panel: F-actin signal in ibw contrast. Red bar indicates PDGF pulse. Time scale: hours: minutes. Scale bars: 10 μm.

Video 6 - Rac1 activation and F-actin dynamics in response to a PDGF pulse

(Related to Fig. 4)

A REF52 cell expressing Lifeact-mCherry (F-actin) and the Rac1-2G biosensor was imaged using epifluorescence microscopy. Left panel: ERs are shown in color code (dynamic range: 1 - 1.19). Right panel: F-actin signal in ibw contrast. Red bar indicates PDGF pulse. Time scale: hours: minutes. Scale bars: 10 μm.

Video 7 - Cdc42 activation and F-actin dynamics in response to a PDGF pulse

(Related to Fig. 4)

A REF52 cell expressing Lifeact-mCherry (F-actin) and the Cdc42-2G biosensor was imaged using epifluorescence microscopy. Left panel: ERs are shown in color code (dynamic range: 1 - 1.33). Right panel: F-actin signal in ibw contrast. Red bar indicates PDGF pulse. Time scale: hours: minutes. Scale bars: 10 μm.

Video 8 - RhoA activation and F-actin dynamics in response to a Y-27632 pulse

(Related to Fig. 5)

A REF52 cell expressing Lifeact-mCherry (F-actin) and the RhoA-2G biosensor was imaged using epifluorescence microscopy. Left panel: ERs are shown in color code (dynamic range: 1 - 1.29). Right panel: F-actin signal in ibw contrast. Red bar indicates Y-27632 pulse. Time scale: hours: minutes. Scale bars: 10 μm.

Video 9 - Rac1 activation and F-actin dynamics in response to a Y-27632 pulse

(Related to Fig. 5)

A REF52 cell expressing Lifeact-mCherry (F-actin) and the Rac1-2G biosensor was imaged using epifluorescence microscopy. Left panel: ERs are shown in color code (dynamic range: 1 - 1.29). Right panel: F-actin signal in ibw contrast. Red bar indicates Y-27632 pulse. Time scale: hours: minutes. Scale bars: 10 µm.

Video 10 - Cdc42 activation and F-actin dynamics in response to a Y-27632 pulse

(Related to Fig. 5)

A REF52 cell expressing Lifeact-mCherry (F-actin) and the Cdc42-2G biosensor was imaged using epifluorescence microscopy. Left panel: ERs are shown in color code (dynamic range: 1 - 1.29). Right panel: F-actin signal in ibw contrast. Red bar indicates Y-27632 pulse. Time scale: hours: minutes. Scale bars: 10 µm.
